# Establishiment of molecular markers for early selection of embryogenic cultures with high embryogenic potential in brazilian pine (*Araucaria angustifolia* (BERT) O. KTZE)

**DOI:** 10.1186/1753-6561-5-S7-P138

**Published:** 2011-09-13

**Authors:** Leonardo Jo, Andre Luis Wendt dos Santos, Paulo Sérgio Schlögl, Miguel Pedro Guerra, Maria Magdalena Rossi, Eny Iochevet Segal Floh

**Affiliations:** 1 Laboratory of Plant Cell Biology, Department of Botany, University of São Paulo, São Paulo-SP, Brazil; 2 Plant Developmental Physiology and Genetics Laboratory, Department of Plant Science, Federal University of Santa Catarina, Florianópolis-SC, Brazil; 3 Laboratory of Plant Molecular Genetic, Department of Botany, University of São Paulo, São Paulo-SP, Brazil

## Background

Brazilian pine (*Araucaria angustifolia* (Bert) O. Ktze) is the only native conifer species with economic importance in Brazil. Recently, due to intensive exploitation Brazilian pine was included in the official list of endangered Brazilian plants. Biotechnology tools, like somatic embryogenesis (SE), may become a potentially useful tool for mass clonal propagation and *ex situ* conservation of commercial and endangered plant species, especially conifers. SE involves the coordinated execution of four steps (embryogenic culture (EC) induction, proliferation, maturation, and plant regeneration). As observed for other conifers, the presence of well-developed early somatic embryos (SE) in EC of Brazilian pine can be considered the pre-requisite for embryo maturation in a medium supplemented with abscisic acid (ABA) and osmotic agents. However, in some genotypes even the presence of bipolar SE does not guarantee embryo maturation. Since SE morphology cannot be used as the only factor for EC selection, the development of molecular markers for early detection of embryogenic cultures responsive to maturation promoters (ABA and osmotic agents) is highly desirable. Polyamines (putrescine (Put), spermidine (Spd), and spermine (Spm)) have been classified as plant growth regulators and hormonal second-messengers playing a critical role in various growth and developmental processes in plants, such as the differentiation and development of somatic embryos. The relation Put/Spd has demonstrated the best answers for predicting embryogenic potential in different plant species. Apart from polyamines quantification, the analysis of gene expression has been used to detect the expression of embryogenesis regulating genes like somatic embryogenesis receptor kinase (SERK), wuschel-related WOX (WOX), and ABA insensitive-1 (ABI1) during conifer somatic embryogenesis. In order to develop molecular markers for early detection of EC with high embryogenic potential in Brazilian pine, we measured the polyamine content (free and conjugate) and the expression of three embryogenesis-regulating genes (SERK, WOX and ABI1) during the proliferation phase of ECs with different maturation capabilities.

## Methodology

For induction of ECs, immature zygotic embryos were inoculated in MSG medium free of growth regulators supplemented with 1.46 g L^-1^ filter-sterilized L-glutamine, 30 g L^-1^ sucrose, 3 g L^-1^ Gelrite® (Sigma) in the dark at 25 ±2°C. After one year of EC proliferation, maturation tests were performed using MSG semi-solid medium supplemented with 120 mM abscisic acid, 9% (w/v) maltose, 7% (w/v) PEG 4000, 3% (w/v) sucrose, and 0.15% (w/v) active charcoal. All ECs were maintained in the dark at 25 ±2°C and subcultured every four weeks by transferring ECs to fresh maturation medium. Total RNA from ECs with different maturation capabilities (0.3 g fresh weight) were extracted with Trizol® (Invitrogen, Carlsbad, CA). cDNA was synthesized using 2 μg of total RNA, digested with DNAse I (Fermentas), and reverse transcribed with 500 ng oligo-dT25-ancored primer (5'-T(25)VN-3') using the High Capacity cDNA Reverse Transcription kit (Applied Biosystems). Primers were designed from nucleotide sequences of Brazilian pine cloned cDNA fragments. The template cDNA were synthesized and the dilutions adjusted with Ubiquitin 1 as an endogenous normalization factor. PCR reactions were carried out in a final volume of 25 µL and the PCR products had an average length of 175 - 200 bp. The RT-PCR products were resolved on 2% (w/v) agarose gels stained with ethidium bromide and photographed.The methodology for the determination of free PAs and conjugate was based on that developed by [1]. Samples (0.2 g fresh weight) were ground in perchloric acid (PCA) 5%. The conjugated forms of the PAs were obtained by hydrolysis (18 h at 110°C) in 12 N chloridric acid (HCl). The samples were then derivatized using dansyl chloride and partitioned with toluene. PAs levels were obtained by means of HPLC using a C18 reverse phase column.

## Results and discussion

Despite of the maturation capability and as observed in other conifer species, the levels of free PAs in all Brazilian pine ECs tested were higher than the conjugated form, and the most abundant PA found was Put followed by Spd and Spm. However, ECs responsive to maturation conditions (with development of mature somatic embryos) showed significantly lower Put/Spd ratios, when compared to non-responsive ECs. A similar profile was observed in embryogenic cultures of *Oryza sativa* L. [2]. In somatic embryos of *Vitis vinifera*, an abnormal growth and a disorganized cellular proliferation were associated to an inadequate Put/Spd ratio [3].

Concerning gene analysis, only the expression of ABI1 gene could be detected during proliferation phase of the ECs cultures. Although ABI-1 gene is normally associated to events mediated by ABA [4], both ECs responsive or not to ABA showed the expression of ABI1. No expression of SERK and WOX could be detected during the proliferation phase of ECs tested, although the expression of these genes was already detected in somatic embryos, late stage zygotic embryos and seedlings of Brazilian pine.

## Conclusions

Based on our results, we can suggest that the Put/Spd ratio can be used as a molecular marker for early selection during proliferation phase of Brazilian pine ECs with high embryogenic potential. However, selected embryogenesis regulating genes (ABI1, SERK-1, and WOX) did not show any association with the embryogenic potential in the ECs tested.
